# 

**Published:** 1909-11-15

**Authors:** 


### ANNOUNCEMENTS.

MICHIGAN FIRST DISTRICT DENTAL SOCIETY. The program for the season will cover eight meetings. Three of the eight will be devoted to clinics exclusively and five to papers and demonstrated lectures. This will insure a good variety and as good men for the lectures and essays are already engaged, a busy and prosperous year's work will undoubtedly be accomplished. The first meeting was held October 14th and Dr. R. H. Varney read a most excellent paper on Syphilis which we print in full in this issue. The November meeting will be a clinic meeting. This is a live society and its social as well as its professional features are most instructive and entertaining.

MICHIGAN DENTAL SOCIETY. The annual meeting was held at Kalamazoo Jnne 29th to July 1st. The meeting, was largely attended and full of interest. The following officers were elected for the ensuing year: President, Dr. George H. Copp; Vice President, Dr. Wm. A. Griffin; Secretary, Dr. Don M. Graham; Tteasurer, Dr. J. W. House. The next meeting will be held in Detroit, June 21 to 23, 1910.

OHIO STATE DENTAL SOCIETY. The forty-second annual meeting of the Ohio State Dental Society will be held in the Southern Hotel, Columbus, on December 7th-9th. This promises to be one of the very best meetings in the history of this society. The program contains the names of such men as Drs. M. L. Rhein, I. N. Broomell, Marcus Ward, C. P. Pruyn and Sidney Rauh, of Cincinnati. The president, Dr. W. H. Whitslar, will give a steriopticon lecture on "The Human Hand." Dr. Whitslar has talked elsewhere on this subject and is a known authority on Palmistry.

The clinic program is the longest in the history of this society.

The arrangements committee will provide a special feature for the entertainment of the members and guests, giving all an opportunity to become better acquainted.

Many new members have been added through the organization of component societies.

Let every member come and bring a friend. A royal welcome and good time awaits you.

F. R. CHAPMAN, *Secy.*

